# Characterization of Carbohydrates, Amino Acids, Viscosity, and Antioxidant Capacity in Rice Wines Made in Saitama, Japan, with Different Sake Rice

**DOI:** 10.3390/foods12214004

**Published:** 2023-11-01

**Authors:** Yutaka Inoue, Sae Ueda, Takashi Tanikawa, Aiko Sano, Ryuichiro Suzuki, Hiroaki Todo, Yuji Higuchi, Kenichi Akao

**Affiliations:** 1 Laboratory of Nutri-Pharmacotherapeutics Management, Faculty of Pharmacy and Pharmaceutical Sciences, Josai University, 1-1 Keyakidai, Sakado 3500295, Saitama, Japan; 2 Laboratory of Natural Products and Phytochemistry, Faculty of Pharmacy and Pharmaceutical Sciences, Josai University, 1-1 Keyakidai, Sakado 3500295, Saitama, Japanryu_suzu@josai.ac.jp (R.S.); 3 Laboratory of Pharmaceutics and Cosmeceutics, Faculty of Pharmacy and Pharmaceutical Sciences, Josai University, 1-1 Keyakidai, Sakado 3500295, Saitama, Japan; 4 Applicative Solution Lab, JASCO Corporation, 2967-5 Ishikawa-machi, Hachioji 1928537, Tokyo, Japan

**Keywords:** rice wine, carbohydrate, amino acid, principal component analysis, antioxidant

## Abstract

We investigated the physicochemical properties of Japanese rice wines, including their functional properties and carbohydrate and amino acid content in solution and solid state. Three samples were tested. The glucose, allose, and raffinose contents in samples (A, B, C) in g/100 g were (3.47, 3.45, 7.05), (1.60, 1.63, 1.61), and (2.14, 2.75, 1.49), respectively. The total amino acid in µmol/mL was (3.1, 3.5, 4.4). Glutamic acid, alanine, and arginine varied in content across the samples. The viscosity (10 °C) and activation energy (ΔE) calculated using the Andrade equation were (2.81 ± 0.03, 2.74 ± 0.06, 2.69 ± 0.03) mPa-s and (22.3 ± 1.1, 22.0 ± 0.2, 21.3 ± 0.5) kJ/mol, respectively. Principal component analysis using FT-IR spectra confirmed the separation of the samples into principal components 2 and 3. The IC_50_ values from the DPPH radical scavenging test were (2364.7 ± 185.3, 3041.9 ± 355.1, 3842.7 ± 228.1) µg/mL. Thus, the three rice wines had different carbohydrate and amino acid contents, viscosities, and antioxidant capacities.

## 1. Introduction

Rice wine in Japan and other Asian nations is traditionally produced from the hydrolysis products of starch and polysaccharides present in the rice cultivated in countries such as China, South Korea, Thailand, the Philippines, and Vietnam [1]. One type of rice wine produced in Japan is sake, which has a tradition of more than 1300 years and is popular as a national drink [2]. Sake manufacturing is a complex process that involves adding koji mold to steamed rice to saccharify the starch, then adding water to form unrefined sake, followed by the addition of yeast for alcoholic fermentation [1]. Sake is rich in nutrients such as sugars, amino acids, organic acids, and aromatic compounds [3] and has a rich taste and flavor. In addition, different varieties of sake rice (the raw material), malted rice, and yeast allow sake to be enjoyed in different ways. However, because sake is an alcoholic product, moderate consumption is recommended.

The adage “A little something to drink is the best medicine” is widely known. Appropriate consumption of alcoholic beverages has been reported to reduce cardiovascular risk by promoting insulin-mediated glucose uptake [4] and increasing HDL and apolipoprotein concentrations [5]. Furthermore, alcohol has an appetite-stimulating effect [6], and drinking sake moderately before or during a meal may help people enjoy their meal and consume the necessary nutrients.

Various naturally occurring carbohydrates are present in sake [7,8]. The monosaccharide d-glucose is present in the body as blood sugar and is a source of energy for physical activity [9]. d-Alose, a rare sugar that acts as the C-3 epimer of d-glucose, is structurally similar to d-glucose. d-Alose competes with glucose for intestinal absorption via SGLT1, which regulates postprandial blood glucose levels [10]. Therefore, it is not absorbed by GLUT5 and may reduce the increase in blood glucose levels. Raffinose is a sugar composed of galactose, glucose, and fructose, and is widely present in cereals, pulses, vegetables, fruits, and other higher plants as a raffinose family oligosaccharide [11,12]. It improves the balance of the intestinal microflora by promoting the growth of bifidobacteria and lactic acid bacteria in the human intestine and reducing harmful bacteria [13]. Furthermore, sake is a fermented food made from rice and has been reported to contain many components, including amino acids, phenolic compounds [14,15], and kojic acid [16], which have whitening properties. Amino acids are components of the body. They include non-essential amino acids synthesized in the body and essential amino acids obtained from the diet. For example, the branched-chain amino acids valine and leucine are essential amino acids that promote muscle protein synthesis [17], whereas glutamic acid is a non-essential amino acid that acts as a flavor component [18]. Ferulic acid, a phenolic acid, is an antioxidant present in rice [19]. Thus, rice wines contain a variety of nutrients such as carbohydrates, amino acids, and organic acids.

Incidentally, ion chromatography is used to analyze the carbohydrates contained in rice wines [20]. Our laboratory previously reported a method for the determination of carbohydrates in fruit wine using a core-shell column and an electrochemical detector (ECD) [21], which can be used in the brewing sector as a simple and effective method for assessing carbohydrate content.

Amino acid analysis is used to evaluate the amino acid content of proteins in foodstuffs and herbal medicines [22]. Therefore, it is used for evaluating the amino acid content of rice wine. It can also be conjectured that the unique texture of rice wines, such as viscosity, differs depending on the fermentation and production processes.

Recently, several local breweries have produced rice wine. These breweries together form a local industry. A facility for easily assessing the characteristics of locally produced rice can help revitalize the local wine industry by providing it with a health-oriented approach. This is expected to allow people to enjoy rice wines in keeping with traditional Japanese culture while promoting the development of regional wine industries amidst growing health consciousness. In this research, we deliberately chose rice wines from Saitama, a local industry near our university, as our research subject from among the many rice wines available. We thought that university researchers and the local community could collaborate to elucidate the functionality of local ingredients and add value through collaborative research, thereby contributing more to regional revitalization and healthy life expectancy. In this study, carbohydrate, amino acid, viscosity, texture, and antioxidant tests were conducted on three different types of rice wines made from sake rice procured from a brewery in Saitama to examine their characteristics.

## 2. Materials and Methods

### 2.1. Materials

Three types of rice wines were purchased from a brewery in Moroyama (Saitama, Japan) and used as Samples A, B, and C (Table 1) [23]. Special-grade glucose was purchased from Fujifilm Wako Pure Chemical Corporation (Tokyo, Japan). Allose and raffinose were supplied by Matsutani Chemical Industry Co., Ltd. All other reagents (special grade) were purchased from Fujifilm Wako Pure Chemical Corporation (Tokyo, Japan).

### 2.2. Methods

#### 2.2.1. Preparation of Freeze-Dried Material

Samples A, B, and C were prepared at 40 °C and 25 mbar after solvent evaporation of the alcohol (Rotavapor R-215, Buchi, Switzerland) and the resulting solutions were freeze-dried. The freeze-dried samples were used for a DPPH radical scavenging test, FT-IR analysis, ^1^H-NMR, and amino acid measurements.

#### 2.2.2. LC Measurement

An electrochemical detector (ECD: SU-300, DKK-TOA, Tokyo, Japan) was used, with 0.1 mol/L NaOH as the mobile phase, a column temperature of 25 °C, a flow rate of 0.3 mL/min, an AS8020 autosampler (Tosoh), and a sample injection volume of 20 μL. The columns were ion-exchange columns with a core-shell type filler and reacted with amines (S-30/70=St (styrene)/DVB (divinylbenzene)-5TMDAH (tetramethyldiaminohexane); φ4.6 mm × 150 mm as described by Yoshimura [21]. The theoretical plate numbers (N) of the samples were determined using the internal processing program of the system. Standard solutions were prepared by weighing approximately 20 mg each of glucose, allose, and raffinose and preparing a 200 µg/mL solution using 100 mL of distilled water. Each concentration (1.25 µg/mL, 2.5 µg/mL, 5 µg/mL, 10 µg/mL, 20 µg/mL, and 40 µg/mL) was then prepared for calibration curve measurements.

A sample solution of 5 g of each rice wine was weighed and diluted 25,000-fold with distilled water to determine the carbohydrate content. As evaluation of the validity of LC measurements, the glucose, allose, and raffinose levels were quantified using standard solutions and evaluated by calculating the linearity as well as the square of the correlation coefficient (R) of the calibration curve for each sample (R^2^) (*n* = 12).

The limits of detection (LOD) and quantification (LOQ) were calculated. Reproducibility and precision were assessed using the relative standard deviation (RSD) with known concentrations of glucose, allose, and raffinose, with each measured repeatedly for *n* = 12. The LOD and LOQ were calculated using the following equations:

(1) LOD=3.3×(s/a)

(2) LOQ=10×(s/a)

s: SD of the intercept for the calibration curve

a: Slope of the calibration curve

#### 2.2.3. Determination of Amino Acids

Freeze-dried Samples A, B, and C were dissolved in 10 mL of distilled water, filtered through a 0.45 µm filter, and diluted 2-fold with buffer for amino acid analysis as the measurement sample. Amino acids were determined using a JLC-500/V instrument (JEOL Ltd., Tokyo, Japan).

#### 2.2.4. Sugar Content Determination

The sugar contents of Samples A, B, and C were measured using a Master-M (Atago Co., Ltd., Tokyo, Japan) at 25 °C (*n* = 3).

#### 2.2.5. pH Measurement

The pH values of Samples A, B, and C were measured using a Horiba pH Meter F-51 (Tokyo, Japan) (*n* = 3).

#### 2.2.6. Surface Tension Measurement

The sample solutions were prepared using 5 mL each of Samples A, B, and C, weighed in 3 cm-diameter Petri dishes. Measurements were performed using a DY500 High Performance Surface Tensiometer (Kyowa (Dyne Master, Kyowa Interface Science Co., Ltd., Saitama, Japan)) at 25 °C (*n* = 3). In addition, a 15% ethanol solution and distilled water were used as reference samples.

#### 2.2.7. Viscosity Measurement

Samples A, B, and C (10 mL each) were weighed in a measuring cup to prepare the sample solution. The temperature was gradually increased from 10 °C to 40 °C, and the change in viscosity was measured. The measurements were performed using an SV-10/SV100 (A&D, Tokyo, Japan) (*n* = 3). The viscosities of a 15% ethanol solution and distilled water were measured as references. The measured viscosity curve was used to calculate the activation energy using the Andrade equation (Equation (3)).

(3) $$ln\eta =lnA+\frac{\Delta E}{RT}$$

H: Viscosity (mPa·s)

A: Ordinary number for viscosity

ΔE: Apparent activation energy (kJ)

R: Gas constant (8.31 J/K·mol)

T: Absolute temperature (K)

#### 2.2.8. Fourier Transform Infrared (FT-IR) Absorption Spectrum Measurements and Principal Component Analysis

FT-IR was performed under the ATR method using a JASCO FT/IR-4600 (JASCO Corporation, Tokyo, Japan) under the following measurement conditions: a wave number of 4000−450 cm^−1^, scanning time of 16 s, and resolution of 4 cm^−1^. IR spectra were used for principal component analysis by the PCA program (JASCO Corporation, Tokyo, Japan).

#### 2.2.9. DPPH Radical Test

A 50 µM solution of 2,2-Diphenyl-2-picry-hydrazyl (DPPH) dissolved in methanol and each sample at different concentrations were added to a microplate at a volume ratio of 1/1 (50 µL DPPH solution and 50 µL distilled water/100 µL sample). The control was 100 µL of water/methanol (1/1) added instead of the sample. Incubation was carried out for 5 min at 37 °C and light-shielded using a Spectra Max microplate reader (Molecular Devices), and absorbance was measured at a wavelength of 517 nm. The inhibition percentage was calculated from the absorbance obtained and the 50% inhibition concentration was determined. The methanol/DPPH methanol solution mixture (1/1) was considered 0% radical removal (A_0_) and the water/methanol (1/1) mixture was considered 100% radical removal (Br: blank). The rate of DPPH radical scavenging activity was calculated using the following equation:

(4) $$Radical\;scavenging\;activity=[1-(As-Br)/(A_{0}-Br)]\times 100$$

#### 2.2.10. Statistical Analysis

Data are expressed as the mean ± standard deviation (SD). Comparisons between experimental groups were assessed with the Tukey test, a one-way ANOVA multiple comparison test with *p* < 0.01, *p* < 0.05 for significant differences due to Statcel—the Useful Addin Forms on Excel, 4th ed.

## 3. Results and Discussion

### 3.1. Confirmation of Conditions for Determination of Sugars Using ECD

Carbohydrate and amino acid content are useful benchmarks for health-conscious consumers when selecting rice wines. Therefore, we measured the glucose, allose, and raffinose levels in Samples A, B, and C. Representative chromatographs of glucose, allose, and raffinose are shown in Figure S1, with specific peaks at retention times of 13.51 min, 15.58 min, and 19.26 min for glucose, allose, and raffinose, respectively.

### 3.2. Validity of LC Measurement

The linearity of the calibration curves for each concentration of glucose, allose, and raffinose and the corresponding correlation coefficient (R) were calculated (Table S1). The LOD and LOQ were calculated from the calibration curves. The peak separation was evaluated by calculating the resolution (Rs). For reproducibility and precision evaluation, repeated measurements (*n* = 12) were performed, and the relative standard deviations (RSD) were calculated. The calibration curves for glucose, allose, and raffinose had R^2^ values of 0.998, 0.999, and 0.999, respectively, with good linearity. The LOD (s/a = 3.3) and LOQ (s/a = 10) were glucose: 0.29 ng/mL and 0.89 ng/mL, respectively; allose: 0.24 ng/mL and 0.72 ng/mL, respectively; and raffinose: 0.68 ng/mL and 2.07 ng/mL, respectively.

### 3.3. Determination of Carbohydrates in Each Rice Wine

The sugar content of each rice wine is listed in Table 2. The glucose contents in Samples A, B, and C were 3.47 g/100 g, 3.45 g/100 g, and 7.05 g/100 g, respectively. Sample C contained approximately twice the amount of glucose compared to Samples A and B. The allose contents in Samples A, B, and C were 1.60 g/100g, 1.63 g/100g, and 1.61 g/100g, respectively. The raffinose contents in Samples A, B, and C were 2.14 g/100g, 2.75 g/100g, and 1.49 g/100g, respectively, indicating that Samples A and B contained approximately 1.5 times more raffinose than Sample C. Allose is generally known for its antioxidant [24], anti-inflammatory [25], and antitumor effects [26]. Raffinose has been reported to balance the intestinal microflora in humans [12,13] and inhibit biofilm formation by the Streptococcus species in the human oral cavity [27]. Thus, allose and raffinose have health benefits. Regarding blood glucose levels, although glucose, a monosaccharide, works to raise and maintain blood glucose, allose competes with glucose in SGLT1 in the small intestine [10], and raffinose is not broken down from trisaccharides because humans do not have the required digestive enzymes for its breakdown [11]. Thus, the possibility that allose and raffinose may be less likely to raise blood glucose is considered. Therefore, we would consider that Samples A and B may have the potential to raise blood glucose levels more gently than Sample C. Thus, we have confirmed that the differences between rice wines A, B, and C can be evaluated in terms of carbohydrates that contribute to human health. In addition, we have demonstrated in this study that the ECD is a useful and simple device for the determination of carbohydrates.

### 3.4. Amino Acid Analysis

Amino acid analysis is a widely used method for evaluating the amino acid content of proteins in crude and processed foods [28]. Therefore, amino acid measurements were performed for Samples A, B, and C, and the results are shown in Table 3. From this analysis, the total amounts of amino acids in Samples A, B, and C were 3.1 µmol/mL, 3.5 µmol/mL, and 4.4 µmol/mL, respectively. Furthermore, different types of amino acids varied in content in Samples A, B, and C. The various amino acid contents in Samples A, B, and C were glutamic acid (0.25 µmol/mL, 0.28 µmol/mL, and 0.35 µmol/mL); alanine (0.58 µmol/mL, 0.78 µmol/mL, and 0.9 µmol/mL); valine (0.14 µmol/mL, 0.21 µmol/mL, and 0.23 µmol/mL); leucine (0.23 µmol/mL, 0.25 µmol/mL, and 0.32 µmol/mL); histidine (0.01 µmol/mL, 0.04 µmol/mL, and 0.07 µmol/mL); and arginine (0.03 µmol/mL, 0.17 µmol/mL, and 0.47 µmol/mL). Glutamic acid has been reported to contribute to taste and acidity [18], and alanine to acidity and bitterness [28]. Valine and leucine are branched-chain amino acids that comprise muscle proteins and contribute to motor functions. The intake of branched-chain amino acids can help reduce sarcopenia, a disease that causes a decrease in total body muscle mass and muscle strength with aging [29]. Histidine is an essential amino acid that works on the sympathetic nervous system to break down fat by working on nerve cells and is converted to histamine in the body [30]. It is known to exert both hepatoprotective and anti-inflammatory effects [31]. Arginine promotes growth hormone secretion and increases muscle strength [32]. Moreover, it lowers blood pressure through its vasodilating effect [33]. Thus, in terms of flavor, richness, and benefits to the body, Sample C contained more amino acids that are beneficial to health than Samples A and B. GABA, PEA, and MEA are not amino acids. However, we measured GABA, PEA, and MEA in order to see if they might be components that reflect differences in manufacturing methods, fermentation, etc., of each rice wine, but no significant differences were observed. There are several possible reasons for the differences in the type and content of amino acids obtained in this study. These are (1) differences in the type of rice used in the production of rice wine, (2) differences in the production method, (3) differences in amino acid and carbohydrate production due to differences in the yeast used in the rice wine production process, and (4) the variability associated with a fermentation process of rice wine.

### 3.5. Sugar Content, pH, Surface Tension Measurement

The pH (acidity) measurement is considered to be a factor in quality control and the evaluation of differences in the fermentation process. In addition, surface tension and sugar content measurements can be used to help evaluate texture. The sugar content of the wines was measured as a convenient sweetness index. The sugar contents of Samples A, B, and C were 10.3%, 10.0%, and 9.8%, respectively (Table 4). Regarding the carbohydrate content, Sample C had a higher glucose content than Samples A and B. However, the results of the sugar content measurement were considered to indicate the overall sugar content of each sample.

The pH measurements were performed to determine the properties of the sample solutions. The pH values of Samples A, B, and C were 4.05, 4.24, and 4.32, respectively (Table 4). Generally, beverages with a pH value less than 4 can cause acid erosion [34]. Alcoholic beverages such as plum wine and wine have a pH less than 4 and may dissolve teeth [35]. All three samples of rice wine, A, B, and C, had a pH of 4 or higher, suggesting that daily consumption of rice wine is unlikely to cause dental caries due to its acid content. Since the pH of each rice wine was approximately 4.0, it was inferred that bacterial growth was suppressed and the acidity level due to fermentation was comparable.

Surface tension measurements were performed to study the variation of viscosity across the sample solutions (Table 4). The surface tensions of the samples were A: 43.20 mN/m; B: 44.08 mN/m; C: 43.90 mN/m; 15% ethanol: 43.35 mN/m; and distilled water: 71.42 mN/m. The surface tension of each sample was lower than that of distilled water due to the weakening of the hydrogen bonds between the water molecules in the aqueous solution. Samples A, B, and C exhibited lower surface tension values than 15% ethanol, and Sample A showed lower values than Samples B and C. This suggests that the composition of the rice wines lowered the surface tension and that the differences among the samples were due to differences in their compositions. Although it was thought that sugar mass affected surface tension, no relationship was observed between sugar mass and surface tension.

### 3.6. Viscosity Measurement

**Figure 1:**
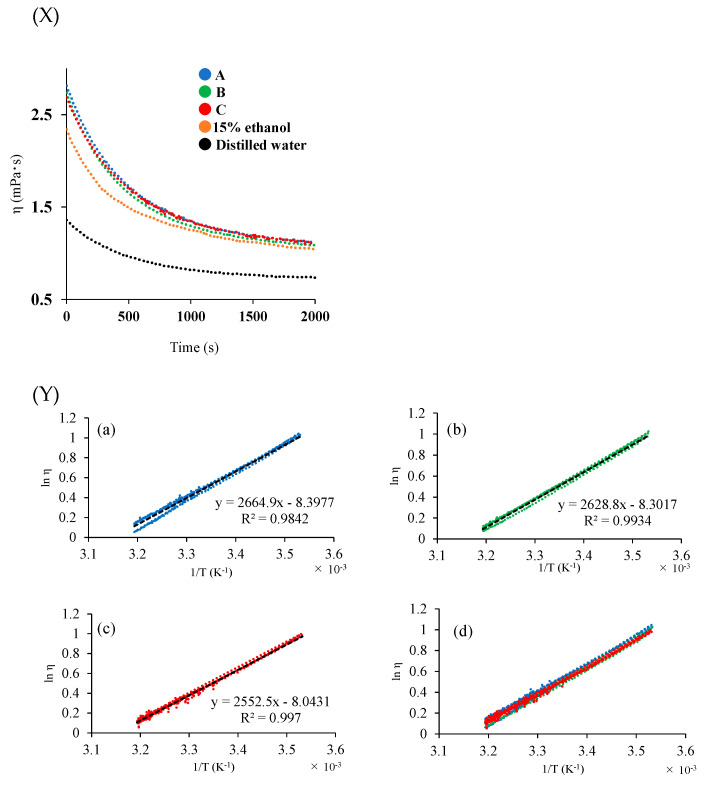
Viscosity depends on temperature increase (**X**). The logarithmic value of viscosity (**Y**) on the vertical axis and the temperature on the horizontal axis of Samples A, B, and C. (**a**) A, (**b**) B, (**c**) C, and (**d**) A, B, C systems.

Viscosity measurement is considered to confirm the difference in texture. The change in the viscosity of each sample solution with temperature was measured (Figure 1X), and the apparent activation energy (ΔE) was calculated using the Andrade equation. The viscosities of Samples A, B, and C at 10 °C were 2.81 ± 0.03 mPa·s, 2.74 ± 0.06 mPa·s, and 2.69 ± 0.03 mPa·s, respectively, and those of 15% ethanol and distilled water were 2.34 ± 0 mPa·s and 1.36 ± 0.03 mPa·s, respectively. The higher viscosity values of the wine samples were due to hydrogen bonding between alcohol and water. The value for Sample A was higher than that for Sample C, probably because of stronger intermolecular interactions in the solution caused by the included components. Thus, the Andrade equation, which expresses the relationship between viscosity and temperature, was used to evaluate the quality of the biofuels. The values of ΔE for the wine samples obtained from the Andrade equation are useful for quantifying the internal structural changes in solution and evaluating Japanese sake (rice wine) [36]. To calculate ΔE from the Andrade equation, a graph of the logarithm of viscosity as a function of the reciprocal of absolute temperature was plotted (Figure 1Y). The ΔE values for Samples A, B, and C, calculated from the Andrade equation, were 22.3 ± 1.1 kJ/mol, 22.0 ± 0.2 kJ/mol, and 21.3 ± 0.5 kJ/mol, respectively. The slope of the straight line corresponding to Sample C is more gradual than that for Samples A and B. Sample C showed a lower ΔE value than A and B even when the temperature changed from 10 °C to 40 °C. This suggests that wine quality is less likely to change with temperature. Moreover, the differences in the internal structural changes in the rice wine solutions were evident.

### 3.7. ^1^H-NMR Measurement

**Figure 2:**
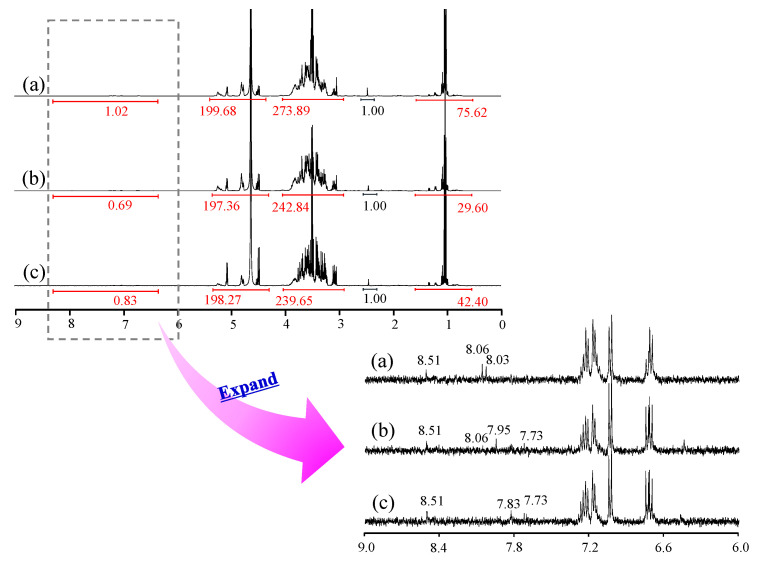
^1^H-NMR (D_2_O) spectra of Samples A, B, and C. (**a**) A, (**b**) B, (**c**) C.

Generally, the spectral area obtained by ^1^H-NMR measurement is proportional to the number of protons, which is used for qualitative analysis. We measured ^1^H-NMR to comprehensively analyze the components and contents of each rice wine (Figure 2). Signals around 1 ppm were attributed to branched-chain amino acids, around 3–5 ppm to carbohydrates, amino acids, and organic acids, and around 7–8 ppm to aromatics. In the 7–8 ppm range, signals were observed at A: 8.03, 8.06, and 8.51 ppm; B: 7.73, 7.95, 8.06, and 8.51 ppm; and C: 7.73, 7.83, and 8.51 ppm, confirming the presence of various aromatic compounds in these samples. The signal area at 2.45 ppm was taken to be 1, and the integral ratios at each chemical shift for A, B, and C around 1 ppm were 75.62, 29.60, and 42.40, respectively; at 3–5 ppm, they were 473.57, 440.2, and 437.92, respectively; and at 7–8 ppm, they were 1.02, 0.69, and 0.83, respectively. Although fragmentary, the NMR spectra revealed differences in the contents of carbohydrates, amino acids, organic acids, and aromatic compounds in the samples.

### 3.8. Fourier Transform Infrared (IR) Absorption Spectroscopy

**Figure 3:**
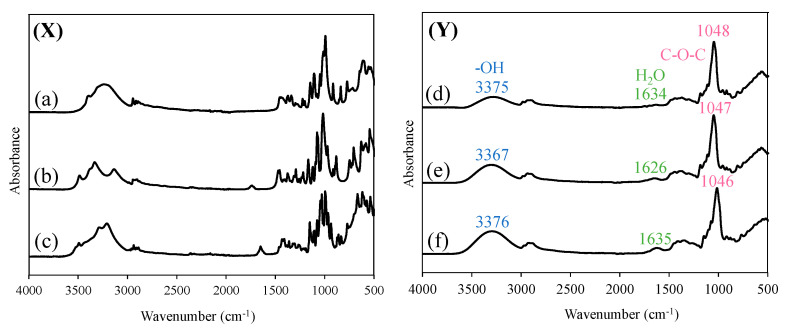
FT-IR spectra of (**X**) (a) glucose, (b) allose, (c) raffinose, and (**Y**) (d) A, (e) B, (f) C.

FT-IR spectra are useful for evaluating the attributes of functional groups in foods, herbal medicines, or other products, and assessing the quality of the products. Therefore, FT-IR measurements of glucose, allose, and raffinose in Samples A, B, and C were conducted using their freeze-dried samples to comprehensively analyze the components contained in each rice wine (Figure 3). The following peaks were observed from the measurement of sugars alone: glucose: 3394, 3303, 1148, 1111, 1078, 1050, 1023, 997, 916, and 839 cm^−1^; allose: 3489, 3379, 3338, 1168, 1123, 1094, 1081, 1031, 947, 896, and 885 cm^−1^; and raffinose: 3943, 3294, 3220, 1148, 1094, 1075, 1049, 1031, 999, 966, 938, 875, 861, and 833 cm^−1^, respectively. The peaks derived from the -OH group in Samples A, B, and C were identified at 3375 cm^−1^, 3367 cm^−1^, and 3376 cm^−1^, respectively. The peaks derived from water in Samples A, B, and C were observed at 1634 cm^−1^, 1626 cm^−1^, and 1635 cm^−1^, respectively. The peaks associated with the C-O-C of sugar for Samples A, B, and C were observed at 1048 cm^−1^, 1047 cm^−1^, and 1046 cm^−1^, respectively. However, because the differences among the samples were difficult to distinguish visually, principal component analysis was performed using the IR spectra of Samples A, B, and C.

### 3.9. Principal Component Analysis (PCA) Using FT-IR Spectra

**Figure 4:**
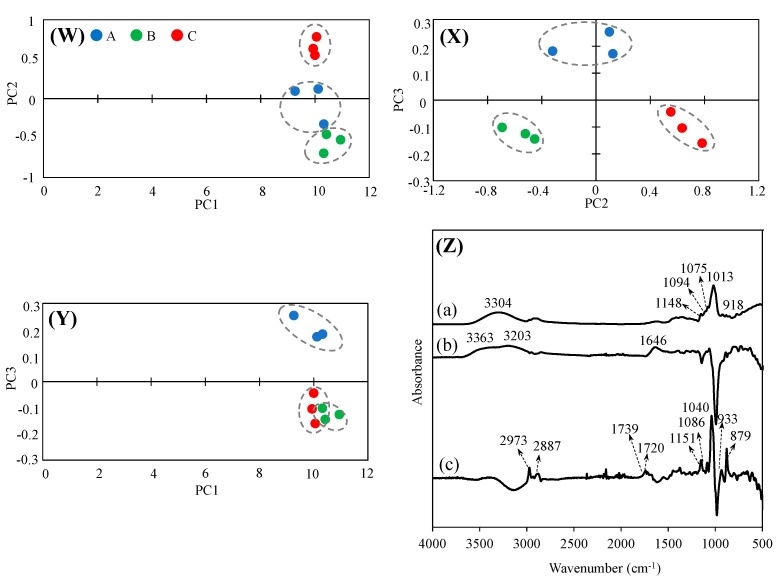
Principal component analysis of A, B, and C using FT-IR spectra score plot of (**W**) PC1 vs. PC2, (**X**) PC2 vs. PC3, (**Y**) PC1 vs. PC3, and FT-IR spectra of (**Z**) PC1, PC2, and PC3. (a) PC1 (b) PC2, (c) PC3.

PCA is used to separate major component spectra from multiple component spectra and to identify components (principal components: PC) that characterize the differences in the spectra. Therefore, a PCA was performed using the IR spectra shown in Figure 4. In the PC1–PC2 plot, A, B, and C were found to cluster differently with PC2 at a value of 10 on the PC1 axis. In the PC2–PC3 plot, A = 0.2, B, and C = −0.1 were distributed around the PC3 axis, confirming the separation of A, B, and C plots by PC2. In the PC1–PC3 plot, no separation of A, B, and C plots was observed in PC1, but in PC3, B and C showed similar clusters; however, the separation was different from that of A. The FT-IR spectra of PC1, PC2, and PC3 are shown (Figure 4Z) to infer which of PC1, PC2, and PC3 were derived from the difference in functional groups. For PC1, a water-derived peak was observed at 3304 cm^−1^, and sugar-derived peaks were observed at 1048, 1094, 1075, and 1013 cm^−1^. In the FT-IR results for sugar alone, these peaks were consistent with those of glucose and raffinose. These findings suggest that although PC1 showed peaks derived from glucose and raffinose, the samples were not separated because of the large influence of water, which is common to all samples. PC2 showed peaks at 3363 cm^−1^ derived from the -OH group, 3203 cm^−1^ derived from the -NH group, and 1646 cm^−1^ derived from the -OH due to water content. Thus, the separation of samples by PC2 in the principal component plot may represent differences in the amino acids and aromatic compounds. PC3 peaks derived from the -CH group were observed at 2973 and 2887 cm^−1^; peaks derived from the C=O group of carboxylic acids were observed at 1739 and 1720 cm^−1^; and peaks derived from sugars were observed at 1151, 1086, 1040, 937, and 879 cm^−1^. The sugar chromatogram confirmed the presence of carbohydrates other than glucose, allose, and raffinose, suggesting the presence of carbohydrates and structures other than glucose, allose, and raffinose in PC3. Foods are mixtures containing a wide variety of substances. Therefore, each component not only has its own taste, texture, etc., but they also interact with each other. Therefore, it is difficult to obtain an “exhaustive” understanding of the characteristics of a food by repeated target analysis, which is the mainstream of food analysis. However, we believe that the combination of FT-IR and PCA presented in this study provides a means to evaluate some of the characteristics of rice wine.

### 3.10. DPPH Radical Scavenging Test

**Figure 5:**
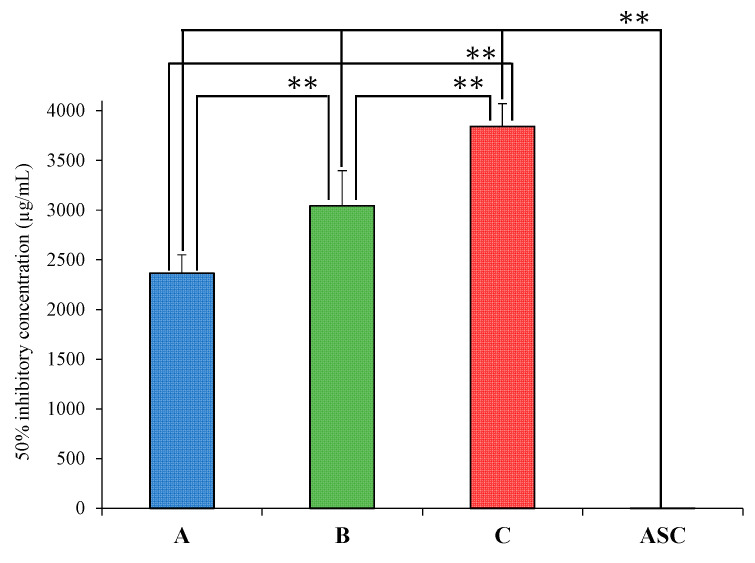
IC_50_ of Samples A, B, C and Ascorbic acid in a DPPH radical scavenging test. Values are the mean ± SD (*n* = 3), ** *p* < 0.01 (Tukey test).

Rice wine is known to contain antioxidants such as ferulic acid [19]. In addition, ^1^H NMR measurements showed differences in the chemical shifts and integration ratios in the aromatic region among the samples, which may also affect their antioxidant capacities. Therefore, a DPPH radical scavenging test was conducted to confirm the antioxidant capacities of Samples A, B, and C. Ascorbic acid (ASC) was tested in the same manner for comparison. The IC_50_ inhibitory concentrations were A: 2364.7 ± 185.3 µg/mL, B: 3041.9 ± 355.1 µg/mL, C: 3842.7 ± 228.1 µg/mL, and ASC: 0.98 ± 0.04 µg/mL (Figure 5). The IC_50_ of Sample A was significantly lower than those of Samples B and C (p < 0.01), and the IC_50_ of Sample B was significantly lower than that of Sample C (p < 0.01). Although the IC_50_ values of Samples A, B, and C were very high compared to the IC_50_ value of ASC, differences in the comprehensive antioxidant capacity were observed. Because sake rice is reported to contain ferulic acid, an antioxidant, and a substance related to beauty [19], this indicates differences in the antioxidant capacities of the samples due to the presence of diverse antioxidants. Hirotsune et al. reported that ethylglucoside suppressed barrier breakdown by promoting keratinocyte differentiation [37]. Wang et al. were the first to investigate the formation of n-propanol, isobutanol, isoamyl alcohol, and phenylethanol during rice wine fermentation. Their group reported differences in higher alcohol content depending on the type of rice used [38]. Xie et al. reported differences in the amino acid content of different rice varieties through rice wine production. They also suggested that the type of rice, i.e., the choice of variety used in production, is more correlated with the quality of rice wine [39]. In this study, substances exhibiting antioxidant activity have not been analyzed in detail. However, the results of the ^1^H NMR and IR measurements and DPPH tests were used to verify the comprehensive antioxidant capacity. Further detailed verification of the components responsible for antioxidant activity should be conducted in the future. In this study, samples of rice wine (Japanese sake) from a sake brewery in Saitama Prefecture, Japan, were used as a case study. Sake has a unique flavor owing to the complex combination of rice, yeast, preparation water, and local temperature. We will continue to evaluate the physical properties of sake through instrumental analysis to provide greater information to people worldwide on what components give rice wine its taste.

## 4. Conclusions

In this study, the differences in the carbohydrate and amino acid contents of rice wines were measured. The results of the viscosity measurements revealed differences in the internal structural changes in the solution state owing to the differences in ΔE calculated from the Andrade equation. Furthermore, ^1^H NMR, IR, and antioxidant tests revealed differences in the substances affecting the antioxidant capacity of rice wine (sake). Finally, differences in rice and production methods were shown to affect the characteristics of rice wine, which is drunk as part of traditional Japanese culture. In other words, the differences between products can be identified by examining the physicochemical properties of rice wine (sake). This study is expected to serve as a foundation for understanding the health aspects of rice wines and boosting the local wine-making industry.

## Figures and Tables

**Table 1:** Raw materials of rice wines A, B, and C [23].

Sample	Alcohol Content (%)	Rice Polishing Ratio (%)	Sake Degree	Amino Acid (mL)	Acidity (mL)	Raw Materials
A	16	60	+1	0.8	1.6	Rice (100% Sake Musashi produced in Saitama), rice koji (produced in Saitama)
B	15	60	+1	0.8	1.4	Rice (100% Yamada Nishiki produced in Saitama), rice koji (produced in Saitama)
C	15	60	+2	1.5	1.4	Rice (produced in Japan), rice koji (produced in Japan)

**Table 2:** Contents of glucose, allose, and raffinose in Samples A, B, and C.

Sample	Glucose (g/100g)	Allose (g/100g)	Raffinose (g/100g)
A	3.47 ± 0.03 ^##^	1.06 ± 0.03 **^, ##^	2.14 ± 0.04 **^, ##^
B	3.45 ± 0.02 ^φφ^	1.63 ± 0.02	2.75 ± 0.05 ^φφ^
C	7.05 ± 0.04	1.61 ± 0.01	1.49 ± 0.04

** *p* < 0.01: A vs. B, ^##^ *p* < 0.01: A vs. C, ^φφ^ *p* < 0.01: B vs. C.

**Table 3:** Contents of amino acid in Samples A, B, and C.

Amino Acid (µmol/mL)	A	B	C	Amino Acid (µmol/mL)	A	B	C
PEA	0	0.01	0	Ile	0.09	0.10	0.12
Asp	0.12	0.11	0.15	Leu	0.23	0.25	0.32
Thr	0.07	0.05	0.08	Tyr	0.13	0.17	0.19
Ser	0.15	0.11	0.17	Phe	0.08	0.10	0.13
Asn	0.12	0.08	0.12	GABA	0.04	0.04	0.05
Glu	0.25	0.28	0.35	MEA	0.44	0.36	0.38
Gly	0.5	0.53	0.51	Orn	0.02	0.01	0.04
Ala	0.58	0.78	0.9	His	0.01	0.04	0.07
Val	0.14	0.21	0.23	Lys	0.09	0.08	0.14
Cys	0.02	0.03	0.02	Arg	0.03	0.17	0.47
				Total	3.11	3.51	4.44

PEA: Phosphoethanolamine, GABA: Gamma-aminobutylic acid, MEA: Monoethanolamine.

**Table 4:** Brix, pH, and surface tension of Samples A, B, and C.

Sample	Brix (%)	pH	Surface Tension (mN/m)
A	10.3 ± 0.1 **^,##^	4.05 ± 0.02 **^,##^	43.20 ± 0.33 ^#^
B	10.0 ± 0.0 ^φ^	4.24 ± 0.03 ^φ^	44.08 ± 0.23
C	9.8 ± 0.0	4.32 ± 0.03	43.90 ± 0.33

** *p* < 0.01: A vs. B; ^#^ *p* < 0.05, ^##^ *p* < 0.01: A vs. C; ^φ^ *p* < 0.05: B vs. C.

## Data Availability

The data presented in this study are available on request from the corresponding author.

## Supplementary Materials

The following are available online at https://www.mdpi.com/article/10.3390/foods12214004/s1. Figure S1: The chromatogram of standard substances peaks (X): 13.51, glucose; 15.58, allose; 19.26, raffinose. Calibration curves of sample by ECD (Y): (a) glucose, (b) allose, (c) raffinose. Table S1: Validation of methods.

## Abbreviations

FT-IR	Fourier Transform Infrared
DPPH	2,2-Diphenyl-2-picry-hydrazyl
SGLT1	Sodium/glucose cotransporter 1
GLUT5	Glucose transporter 5
ECD	Electrochemical detector
LOD	Limit of detection
LOQ	Limit of quantification
RSD	Relative standard deviation
ATR	Attenuated Total Reflection
PCA	Principal component analysis
