# Supplementary material for: Characterization of Carbohydrates, Amino Acids, Viscosity, and Antioxidant Capacity in Rice Wines Made in Saitama, Japan, with Different Sake Rice
Source: Foods. 2023 Nov 1;12(21):4004. doi: 10.3390/foods12214004 (PMC10647497; doi:10.3390/foods12214004)
Supplement: Supplementary file 1 [file foods-12-04004-s001.zip › foods-2628923-supplementary.pdf]

foods

MDPI

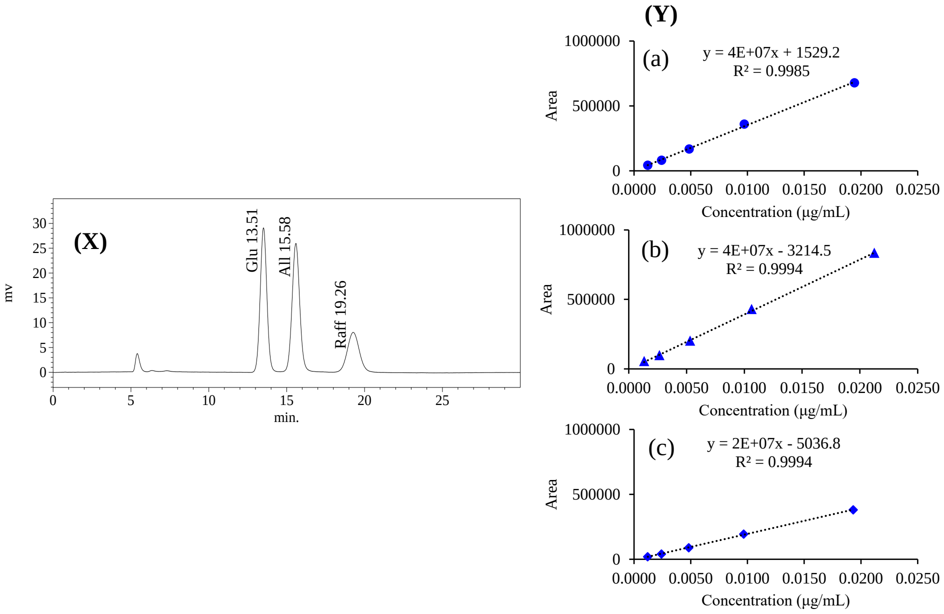

Figure S1. The chromatogram of standard substances peaks (X). 13.51; Glucose, 15.58; Allose, 19.26; Raffinose. Calibration curves of sample by ECD. (Y): (a) Glucose, (b) Allose, (c) Raffinose.

Table S1. Validation of the methods.

Standard substance	R^2	Rs	RSD (%)	LOD (ng/mL)	LOQ (ng/mL)
				3.3s/a	10s/a
**Glucose**	**0.998**	**2.10**	1.88	0.29	0.89
Allose	0.999	2.62	1.12	0.24	0.72
Raffinose	0.999	4.35	1.90	0.68	2.07

s: SD of absorbance of blank sample (n=12). a: The slope of the calibration curve near the detection limit.
